# Reply to Beilstein, C.M.; Wuethrich, P.Y. Comment on “Crettenand et al. Is Continuous Wound Infiltration a Better Option for Postoperative Pain Management after Open Nephrectomy Compared to Thoracic Epidural Analgesia? *J. Clin. Med.* 2023, *12*, 2974”

**DOI:** 10.3390/jcm12185918

**Published:** 2023-09-12

**Authors:** François Crettenand, Nady Assayed-Leonardi, Felix Rohrer, Silvia Martinez Carrique, Beat Roth

**Affiliations:** 1 Department of Urology, Lausanne University Hospital and University of Lausanne, Rue du Bugnon 46, 1011 Lausanne, Switzerland; 2 Department of Anesthesiology, Lausanne University Hospital and University of Lausanne, Rue du Bugnon 46, 1011 Lausanne, Switzerland

We appreciate your comprehensive comment [1] on our study, “Is continuous wound infiltration a better option for postoperative pain management after open nephrectomy compared to thoracic epidural analgesia?” recently published in JCM [2].

According to your citation of Joshi and Kehlet, thoracic epidural analgesia (TEA) is the gold standard for major thoracic or abdominal surgery, but their recommendations for TEA on behalf of the Procedure-Specific Postoperative Pain Management (PROSPECT) working group appears to be limited [3]. They suggest that the role of TEA within a true ERAS^®^ programs is on the decline due to concerns about potential adverse effect, such as delaying ambulation and discharge. We agree that epidural analgesia might still have a role in certain high-risk cases, but the overall trend of ERAS^®^ protocol seems to be moving towards alternative analgesic techniques that balance pain relief with the goal of early recovery and ambulation. At this point, our study—though retrospective in nature and in full awareness that a prospective randomized study is necessary for high-evidence conclusions—adds novel information on possible benefits of continuous wound infiltration (CWI), as there is no higher-degree evidence comparing CWI versus TEA for open nephrectomy to the best of our knowledge.

As stated in our methodology, data of this study were retrieved from the ERAS^®^ International Audit System (EIAS). Unfortunately, information is pre-defined and does not contain every interesting variable for different research topics. Nevertheless, the advantage of this databank is to provide reliable data, as the information is prospectively entered by a trained and dedicated “ERAS^®^” nurse. Moreover, there is a constant auditing process further adding to data quality. We thank the authors for proposing a patient-centered outcome measure, which would have added valuable information. Still, by including nausea, pain scores and mobilization in our analysis, we address at least a portion of the QoR-15 items [4].

We thank you for the opportunity to specify some issues, notably on the pain scores used. The reported pain scores in this study correspond to the maximal pain score documented, most probably during mobilization. This explains the higher pain scores in the CWI group while patients were presumably mobilized. Due to the ERAS^®^ protocol setting, it remains, however, purely speculative that the maximal pain score corresponds to the pain score at mobilization. Concerning further effects of TEA, our results showed that the CWI group was mobilized earlier, and patients had less nausea and earlier gut mobility despite the rationale of hemodynamic assessment and visceral analgesia. Nevertheless, the retrospective design does not permit to study the causative link, but the results are similar to the randomized controlled trial investigating continuous transversus abdominal plane (TAP) block versus TEA in donor nephrectomy patients [5]. Although the trial included only 30 patients and the dosage of the TEA seems low with Ropivacaine 0.2% 2 mL/h, pain scores at rest and mobilization were lower in the TAP group, and importantly, catheter removal and ambulation were faster in the TAP group, which was also noticed in our study. One must be aware that this point is crucial and a cornerstone of enhanced recovery after surgery.

Regarding the potential of systemic toxicity of Ropivacaine, several key points emerge from the existing literature. To begin with, the study conducted by Bianconi et al. sheds light on the peak concentration of ropivacaine (C_max_) while using CWI devices [6]. It displayed a range spanning from 0.32 to 1.59 µg/mL. The mean reported C_max_ was 0.71 µg/mL (SD ± 0.11 µg/mL). It is worth noting that the generally accepted threshold for neurological toxicity associated with ropivacaine is commonly cited as being around 2.2 µg/mL [7]. Intriguingly, several investigations have documented instances where C_max_ levels reached up to 3 µg/mL yet failed to induce any discernible toxicity. Particularly noteworthy among these studies is the work by Petterson and colleagues [8]. Furthermore, the research conducted by Pere et al. in 2011 offered insight into pharmacokinetics of ropivacaine in the context of renal failure. Their findings suggest that renal impairment does not significantly impact ropivacaine elimination, largely due to the compensatory effect of non-renal elimination mechanisms [9]. This information has far-reaching implications, indicating that CWI could be employed safely irrespective of renal function.

Moreover, we would like to provide reassurance that according to our local pain management protocol, strong opioid prescriptions are adapted according to renal function, though not stated in our methodology. For a glomerular filtration rate (GFR) between 30 and 60 mL/min/1.73 m^2^, we lower the morphine dosage by 50% or use hydromorphone. If there is a severe renal impairment (GFR < 30 mL/min/1.73 m^2^), we switch to buprenorphine or fentanyl. Furthermore, to compensate for the absence of postoperative renal function in our study, we reexamined our dataset. We found that the mean creatinine value at hospital discharge was 85.7 mmol/L (SD 28.9 mmol/L). The mean GFR was calculated to be 78.9 mL/min/1.73 m^2^. Among these patients, only 3.1% had a severe renal impairment (GFR < 30 mL/min/1.73 m^2^). These values indicate a largely sufficient renal function in general for this type of surgical patient in our series. In summary, these data underscore the potential safety of CWI utilization, even in the presence of varying degrees of renal dysfunction. This serves to fortify our stance that CWI can be safely embraced in clinical practice without precipitating undue toxicity concerns.

As a matter of conclusion, while we acknowledge your concerns about the lack of specific details in the paper supporting the 40% overall cost reduction, we want to clarify our rationale behind this finding. The cost reduction we reported correspond to the true cost as charged to health insurance and considers the assessment of various factors including not only the differences in operating room time but also other resource-related aspects that contribute to the overall cost. While it is true that the difference in total OR time between the two groups may not appear substantial, our analysis considered all additional factors that contribute to cost reduction such as reduced nursing workload, no extensive monitoring for CWI, and reduced postoperative care. We also want to emphasize that our study took place in a specific institutional setting with established protocols and resource allocation practices. The cost reduction may be influenced by unique dynamics of our institution, to date the only true certified ERAS^®^ center for urology in Switzerland [10]. We value the perspective of detailed cost analysis, but this was beyond the scope on this article. It could, however, represent an interesting subject for further studies.

We hope that this clarifies our position to promote CWI and provides a better context for it. We remain committed to communicating our findings and addressing any concerns raised by reviewers.
